# The Patient Navigator: Can a systematically developed online health information tool improve patient participation and outcomes related to the consultation in older patients newly diagnosed with colorectal cancer?

**DOI:** 10.1186/s12885-021-09096-6

**Published:** 2022-01-25

**Authors:** Melanie de Looper, Ellen M. A. Smets, Barbara C. Schouten, Sifra Bolle, Eric H. J. Belgers, Eric H. Eddes, Jeroen W. A. Leijtens, Julia C. M. van Weert

**Affiliations:** 1 Amsterdam School of Communication Research/ASCoR, University of Amsterdam, 1018 Amsterdam, WV Netherlands; 2 Department of Medical Psychology, Amsterdam UMC, University of Amsterdam, Amsterdam, The Netherlands; 3 Amsterdam Public Health research institute, Amsterdam, The Netherlands; 4 Centre for Health and Society, National Institute for Public Health and the Environment (RIVM), Bilthoven, The Netherlands; 5 Zuyderland Medical Center, Sittard, Geleen, The Netherlands; 6 Department of Surgery, Deventer Hospital, Deventer, The Netherlands; 7 Dutch Institute of Clinical Auditing, Leiden, The Netherlands; 8 Laurentius Hospital, Roermond, The Netherlands

**Keywords:** online patient tool, online health information, recall, anxiety, satisfaction, patient participation

## Abstract

**Background:**

Older cancer patients may search for health information online to prepare for their consultations. However, seeking information online can have negative effects, for instance increased anxiety due to finding incorrect or unclear information. In addition, existing online cancer information is not necessarily adapted to the needs of older patients, even though cancer is a disease often found in older individuals.

**Objective:**

The aim of this study was to systematically develop, implement and evaluate an online health information tool for older cancer patients, the Patient Navigator, providing information that complements the consultation with healthcare providers.

**Method:**

For the development and evaluation of the Patient Navigator, the four phases of the MRC framework were used. In the first and second phase the Patient Navigator was developed and pilot tested based on previous research and sub-studies. During the third phase the Patient Navigator was implemented in four Dutch hospitals. In the last phase, a pilot RCT was conducted to evaluate the Patient Navigator in terms of usage (observational tracking data), user experience (self-reported satisfaction, involvement, cognitive load, active control, perceived relevance of the tool), patient participation (observational data during consultation), and patient outcomes related to the consultation (questionnaire data regarding anxiety, satisfaction, and information recall). Recently diagnosed colorectal cancer patients (N = 45) were randomly assigned to the control condition (usual care) or the experimental condition (usual care + Patient Navigator).

**Results:**

The Patient Navigator was well used and evaluated positively. Patients who received the Patient Navigator contributed less during the consultation by using less words than patients in the control condition and experienced less anxiety two days after the consultation than patients in the control condition.

**Conclusion:**

Since the Patient Navigator was evaluated positively and decreased anxiety after the consultation, this tool is potentially a valuable addition to the consultation for patients. Usage of the Patient Navigator resulted in patients using less words during consultations, without impairing patients’ satisfaction, possibly because information needs might be fulfilled by usage of the Patient Navigator. This could create the possibility to personalize communication during consultations and respond to other patient needs.

## Introduction

Patients newly diagnosed with cancer often experience unmet information needs by solely communicating with their healthcare providers [1–3]. For older patients, the risk of unfulfilled information needs is even higher, since age-related cognitive (lower pace of processing information) and sensory declines (problems with their vision and hearing) can hinder the communication with the healthcare provider [4]. Older patients find it more challenging to indicate their information needs during consultations with healthcare providers, participate less during consultations [5] and experience more difficulty with correctly recalling information given during consultations than younger patients [6, 7]. From the healthcare provider perspective, stereotypes and negative attitudes towards older patients might negatively affect the interaction during consultations, for example by providing less information [5]. These above mentioned risks can be problematic because cancer is a disease that often comes with aging [8, 9].

Patients with unfulfilled information needs after a medical consultation can turn to the internet to check the information given by the healthcare provider or to find additional information [3, 10, 11]. Besides, older patients also search for online health information to prepare for consultations with healthcare providers [11–13]. A previous study investigating online health information seeking among middle-aged and older cancer patients showed that more than half of these patients searched online for information regarding their illness or diagnosis before consultations [14].

However, results regarding the effects of patients seeking online for such information are inconclusive. On the one hand, seeking for information online can reassure patients, decrease their anxiety [15], and increase feelings of empowerment and patient participation during consultations [13], satisfaction with the consultation [16] and recall of information provided during the consultation [17]. On the other hand, seeking for information online can have negative effects, because the information patients find online may be incorrect or difficult to understand [18]. Older patients are even more susceptible to these negative consequences, such as increased anxiety [19], than their younger counterparts, as they are more at risk of misunderstanding the information they find online due to age-related declines [20, 21]. Besides, older patients can have specific information presentation preferences because of age-related sensory impairments [22]. For example, patients experiencing hearing loss might prefer visual information. If online health information is not adapted to these preferences, this might contribute to negative outcomes of seeking for online health information.

Another reason for these inconclusive results could be related to the content of the information patients find online. If the online health information converges with information given by healthcare providers during consultations this leads to positive health outcomes [23]. On the contrary, if patients encounter online health information that is not consistent with the information in the consultation this might result in negative consequences, such as confusion. Furthermore, if patients do not discuss the discrepancy between information found online and information given during the consultation, healthcare providers may not be able to adequately address this discrepancy.

Taking these factors into consideration, this study aimed to systematically develop, implement and evaluate an online health information tool for older patients newly diagnosed with cancer; The Patient Navigator, providing information that complements the consultation with healthcare providers. The tool is specifically developed for colorectal cancer patients since this type of cancer is often seen in older individuals, with almost 80% of the individuals diagnosed being older than 65 years old [9]. Features that seem promising for older cancer patients and possibly match their preferences were tested during the development phase. In addition, all information provided in the tool has the function to complement the information given during consultations with healthcare providers and to support patients to take on a more active role during these consultations.

The development and evaluation process of the Patient Navigator was guided by the MRC framework , which offers guidelines for appropriately developing, implementing and evaluating complex health interventions [24]. In the framework four phases are distinguished: Phase 1: Development, Phase 2: Piloting testing, Phase 3: Implementation and Phase 4: Evaluation. In the following paragraphs these phases, and all steps taken in each phase, will be described consecutively.

## Phase 1: Development of The Patient Navigator

### Content

The content of the Patient Navigator was derived from the largest Dutch online platform offering information about cancer (www.kanker.nl). The information on this platform was chosen as a starting point for the Patient Navigator because the information is reliable and checked for correctness and readability by various professional organisations (Dutch Cancer Society, Dutch Federation of Cancer Patient Organizations and the Integrated Cancer Centre Netherlands). Information specifically relevant for colorectal cancer patients was selected to be included in the Patient Navigator and assessed by healthcare providers of hospitals participation in the pilot RCT, who were familiar with what was generally discussed during consultations with colorectal cancer patients and thus could ensure that the information in the Patient Navigator was aligned with the information that was provided during these consultations.

### Presentation of Information Features

Results of previous studies carried out by our research group were taken into account to decide what features should be included in the Patient Navigator. Studies among older adults regarding information presentation mode and additional features to prepare for, or process information given during a consultation will be discussed below.

#### Information presentation mode

First, experimental studies by the research group showed positive effects of adding audio-visual formats to online health information [25–27]. For example, animations [26]. videos in which a patient shared experiences with the viewer [28], and videos in which a healthcare professional explained a treatment [25] positively affected older patients’ satisfaction with the information and information recall (when compared to written text only) Second, illustrations have shown to positively influence the comprehensibility [29] and attractiveness of online health information, and improved information recall among older patients when compared to text only [30, 31].

Lastly, to address the various information preferences of older patients, it is important that the information can be individually tailored, or in other words, adjusted to the specific preferences of each individual patient [33]. The option to tailor the content in online tools was previously shown to increase perceived relevance and improve information processing [33–35]. For this reason, content tailoring was incorporated in the Patient Navigator based on this previous research. In addition, a recent experiment by our research group has shown that the option to self-tailor the information presentation mode of online health information positively influenced the evaluation and information processing of the health information [36, 37]. Especially for older patients, online information with self-tailoring options increased attention and information recall and was perceived as more attractive and comprehensible than information without self-tailoring options [38, 39]. Therefore, it was expected that adding self-tailoring features would positively influences patient outcomes and was therefore included in the Patient Navigator.

#### Additional features

Aside from information presentation mode, other pre- and post- consultation features were considered for inclusion in the Patient Navigator based on results of our previous studies. To help older patients to prepare for consultations with healthcare providers, it might be valuable to add a question prompt list (QPL), i.e., a list of structured questions that patients can ask during consultations [40, 41]. Studies of our group showed that QPL’s are effective in increasing patient participation (especially question asking), during consultations with healthcare providers [42], and to decrease anxiety and improve information recall [40].

Since one of the motivations of older patients to use online health information is to get more certainty regarding the information given by the healthcare provider [10, 11], it can be helpful to integrate an audio facility tool which provides patients with the opportunity to listen back to their recorded consultations. Indeed such a feature was positively evaluated by patients in previous research [42]. An audio facility might be especially helpful for older patients, since they experience difficulties with recalling information given to them in consultations [43].

Based on the above mentioned studies, it was decided to include the following elements in the first prototype of the Patient Navigator: animations showing treatments or procedures, patient perspective narrated videos about their experience with the procedure in the hospital or a specific treatment, videos with healthcare providers explaining treatments and/or procedures, cognitive and affective illustrations to support the textual information, and the option for patients to self-tailor the information mode. With regards to additional pre- and post-consultation features, a set of 14 QPL’s (QPL for each specific treatment or test), a more general list of questions appropriate for every treatment functioning as decision support tool and an audio facility were incorporated.

### Usability

Since we lacked insights regarding usability of online health information tools, older patients’ experiences with the usability of already existing online cancer information tools were investigated by means of a think-aloud study [20]. For more details regarding the method of this study, see appendix 1.

Results showed that older patients appreciated online cancer information tools and experienced these as useful. However, they also experienced difficulties in terms of usability such as navigation through the features and finding information within features that had complex navigation structures. Some of the navigation problems older patients experienced were caused by the lay-out of the features such as small buttons and colours that had low level of contrast. In addition, older patients appreciated information presented in different information provision modes and varied greatly in the amount of information they wished to receive. Older patients also experienced the questions that functioned as decision support as too complex.

Because of older patients’ difficulties with navigation, the navigation of the Patient Navigator was kept simple in the prototype. For web pages of the Patient Navigator which required more navigation, such as the page including the QPL, patients were provided with clear instructions. Lastly, the list of questions functioning as decision support tool was simplified resulting in five questions (i.e. ‘what are the treatment options?’, ‘what are the advantages of each treatment?’, ‘what are the advantages and disadvantages of each treatment?’, and ‘what does each option mean for my daily life?’). Multiple recommendations for the development of the Patient Navigator followed the outcomes of this study. For more details see Bolle et al., 2016 [20].

### Lay-out

Interviews were conducted to determine what colours and illustrations older patients would prefer in an online health information tool. Three male and six female individuals (n=9) were interviewed, with age varying from 67 to 87 and computer experience ranging from not using a computer at all to daily use. The lay-out of a comparable online health information tool was used (the PatientVoice) and twenty-two colour schemes were presented in different environments were tested. For more information regarding the method of this study see appendix 2.

Results showed colours were evaluated more positively than black, white and grey tones. However, darker colours were preferred such as dark purple, dark blue or dark red since brighter colours such as light shades of orange and green were experienced as difficult to read. Regarding visuals, all participants preferred drawn illustrations over photos. For illustrations, contrasting darker colours were preferred as well, especially blue and red shades. Again illustrations in bright colours were perceived negatively and too stressful for the eyes. Based on these results a mood board was created that was presented to the graphical designer to design the lay-out of the Patient Navigator and a first prototype of the Patient Navigator was created.

## Phase 2: Pilot testing phase

The aim of the pilot testing phase was to test and revise the prototype of The Patient Navigator based on a second think aloud study and a survey study assessing usability before establishing a final version.

### Think Aloud Usability Study

The first step of phase 2 involved a think-aloud study in which participants were asked to give their opinion about The Patient Navigator. Participants were recruited via an online panel (PanelCom, a panel founded by the department of Communication Science at the University of Amsterdam and the department of Medical Psychology at the AMC). Participants had to be 65 years or older and (ex-) colorectal cancer patients or partners of (ex-) colorectal cancer patients. In total, twelve participants were included; eight (ex-) colorectal cancer patients and four partners of (ex-) colorectal cancer patients. The participants were asked to interact with the Patient Navigator at home, under observation of two researchers. Half of the participants were moderated by the first researcher (research assistant 1) and observed by the second researcher (research assistant 2), while the other half was moderated by the second researcher (HT) and observed by the first researcher (RK). While interacting with the prototype, participants had to complete five different tasks ranging from simple navigation tasks to more complex search tasks and using the different functions provided in the tool (i.e. explore the Patient Navigator, go to home page, search for treatment information, search for help with preparing for a consultation and a self-tailoring task). Participants were asked to explicitly state their thoughts while interacting with the prototype. In addition, screen recordings were made of participants use of the prototype.

Researchers’ observations and patients’ comments were coded based on a codebook including four categories; navigation (i.e. menu, headers, sub headers), content (i.e. understandability, relevance, completeness), lay-out (i.e. colour scheme, illustrations) and overall satisfaction [45]. Moreover, the search tasks were analysed based on how the task was carried out (task completed with or without help from the researcher, how many pages the participants visited before completing the task, the time it took before the task was completed). The think aloud sessions lasted no longer than one hour.

Results showed that although participants were generally satisfied with the information modality and comprehensibility of the Patient Navigator (for example patients appreciated the combination of text and video), they still experienced difficulties when navigating through the tool in particular with finding the decision support tool and the QPL’s. In addition, the self-tailoring function was not only shown on a page (‘personal page’), but also as a pop-up that appeared right after opening the Patient Navigator, forcing patients to self-tailor the content. The pop-up was disliked by participants as they indicated they wanted to see the content of the Patient Navigator first before deciding on amount and mode of information presented. Regarding the lay-out, participants were not satisfied with the illustrations shown on the homepage and felt the depicted characters (nurse, doctor and patient) were too old-fashioned.

Based on these results, changes in the navigational structure of the Patient Navigator were made. Also, the labels used for various functionalities were adapted to make them more self-explanatory. The self-tailoring pop-up was removed and the self-tailoring function was now only accessible via a separate page. This page was renamed as ‘adjust the website’ instead of ‘personal page’ and an arrow was added accompanied by the text ‘decide what you want to see’. The page where patients could find decision support information was renamed as ‘preparation for the consultation’ and the QPL was relocated to this page. The illustrations were adapted based on the respondents’ feedback to make them less old-fashioned (for example their clothing was changed into a more modern uniform). Other adaptations involved: entire buttons being made clickable (instead of the text only) and some smaller changes in the lay-out of the homepage.

### Survey Usability Study

To test the usability of the second Patient Navigator prototype, an online questionnaire was distributed via PanelCom among people aged 65 years and older (n=72). Participants were asked to use the Patient Navigator and received the questionnaire a few days later. The questionnaire consisted of questions originating from the Website Satisfaction Scale (WSS,[32]) and the Website Evaluation Questionnaire (WEQ, [46]. The WSS measured the attractiveness, comprehensibility and emotional support of the Patient Navigator on a 7-point Likert scale, ranging from totally disagree (1) to totally agree (7). The WEQ measured satisfaction with the content, ease of use and evaluation of the lay-out of the Patient Navigator on a 5-point Likert scale, ranging from totally disagree (1) to totally agree (5). Furthermore, open questions were added to allow participants to elaborate on their answers.

**Fig. 1:**
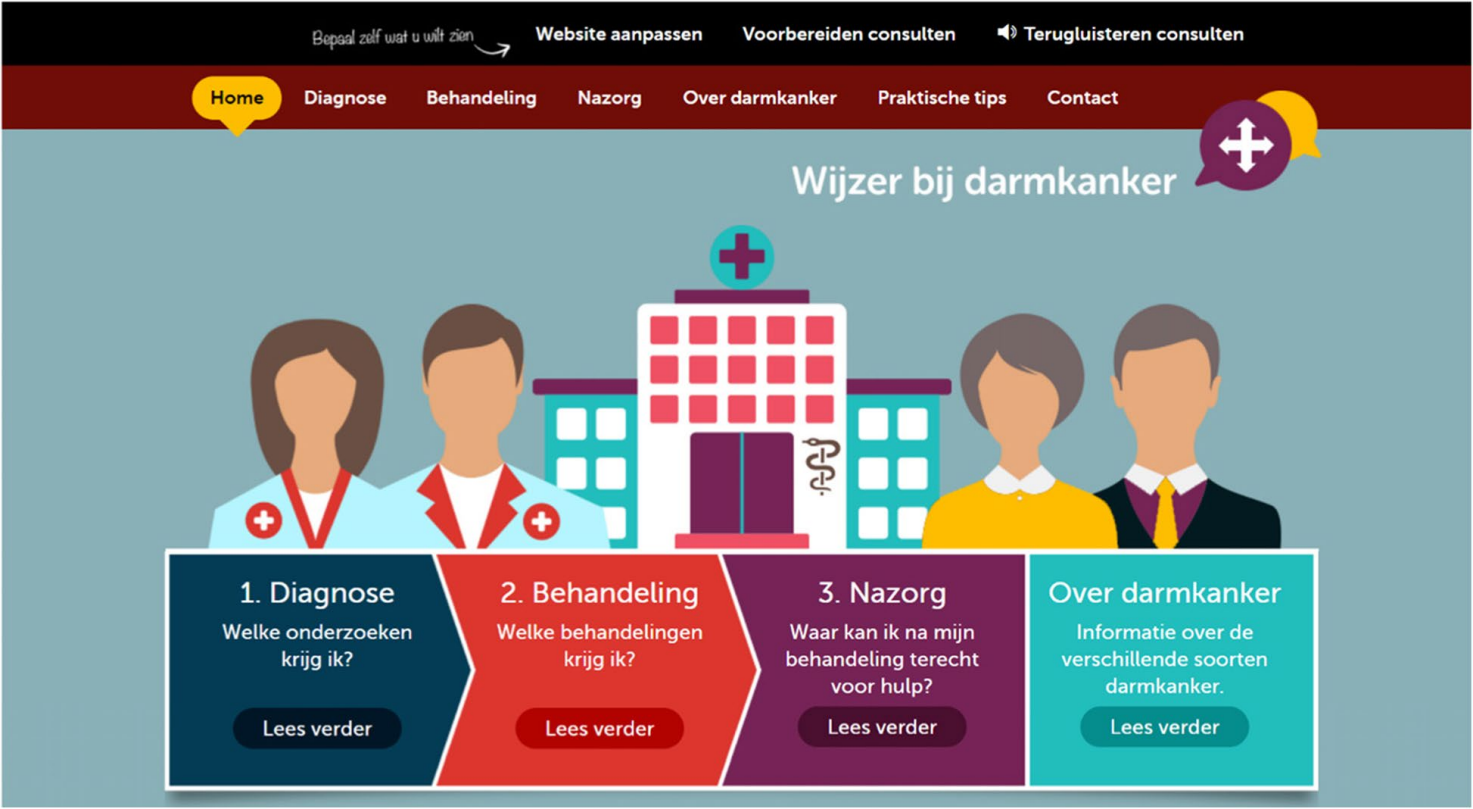
Final version of the Patient Navigator

Results of the WSS showed that the Patient Navigator was evaluated positively regarding its attractiveness (*M*=5.15, *SD* = 1.11) and comprehensibility (*M*=4.35, *SD* = 1.52) and satisfactory regarding emotional support (*M*=3.86, *SD* = 1.42). Besides, based on the WEQ, the relevance (*M*=3.21, *SD* = .48), comprehensibility (*M*=3.33, *SD* = .49), completeness (*M*=3.09, *SD* = .49), ease of use (*M*=3.11, *SD* = .53), navigation (*M*=3.26, *SD* = .58), structure (*M*=3.28, *SD* = .57), speed (*M*=3.05, *SD* = .58) and lay-out (*M*=3.14, *SD* = .60) of the Patient Navigator were also evaluated positively. The answers to the open questions provided guidance for some final, minor adjustments, for example renaming some labels in the menu and adapting the lay-out of the menu shown on the homepage. See final version of the Patient Navigator in Figure 1 and see appendix 3 Table 5 for an overview of al preparatory phases and sub studies

### Intervention – Content of the Patient Navigator

Based on the development and pilot testing phases (phase 1 and 2), the menu header of the Patient Navigator included six main pages: Diagnosis, Treatment, Aftercare, About Colorectal Cancer, Practical Tips, and Contact. On the ‘Diagnosis’ page information about diagnostic tests was presented, for example information about a CT-scan. On the ‘Treatment’ page different treatment options were explained, for example surgery and chemotherapy. The ‘Aftercare’ page contained information about organizations that could help patients during or after their treatment. The information on the ‘About Colorectal Cancer’ page was mainly about different types of colorectal cancer and the different stages of cancer. On the ‘Practical Tips’ page information regarding how to practically deal with the diagnosis was presented, for example advising patients to write down emotions, weigh themselves regularly and keep track of their stool pattern. Lastly, on the ‘Contact’ page, patients could fin contact details of the surgeons and nurses from the hospital they were treated in.

Besides these six main pages, three other features were presented in the Patient Navigator: a self-tailoring feature, a preparation feature including decision support questions and a QPL, and an audio-facility feature.

Patients received the Patient Navigator with all content and modalities visible. They could tailor this to their preferences by decreasing the amount of content and the modality in which the information was presented.

## Phase 3: Implementation

The final prototype version of the Patient Navigator was presented to stomach-liver-intestine healthcare providers (n = 16) of four participating hospitals for feedback. Based on their feedback, final changes were made regarding the content of the Patient Navigator, for example, describing certain treatment options in more detail. Consent about all pages was reached and all participating healthcare providers consented to participating in the pilot RCT. After informing all other staff members of the stomach-liver-intestine departments (i.e. nurses and medical secretaries) about the study and bringing the intervention to their attention, the Patient Navigator was implemented at the stomach-liver-intestine division of the participating hospitals.

## Phase 4: Evaluation

### Design

To evaluate the Patient Navigator, a pilot randomized controlled study was conducted in the four participating hospitals. All methods used were performed in accordance with the relevant guidelines and regulations. Since the Patient Navigator was developed as a tool complementing the information given during the consultation with a healthcare provider in which diagnosis and treatment options are discussed, colorectal cancer patients scheduled for such a consultation with a stomach-liver-intestine surgeon were approached for the pilot RCT. For the following steps of carrying out the pilot RCT, the Cochrane guidelines were followed. This resulted in a computerized randomization of patients to either the control condition or the experimental condition, with healthcare providers blinded for the randomization. Patients in the control condition received the usual care procedure, which was only a consultation with the surgeon, while patients in the experimental condition received the Patient Navigator before the consultation.

Patients’ evaluation of the Patient Navigator was assessed in three ways. First, usage of the Patient Navigator was recorded by a built-in tracking system so that every action on the Patient Navigator was saved (i.e. total time spent on the Patient Navigator and time spent per page, number of visits, number of page visits, number of clicks, usage of self-tailoring function, usage of pre- and post-consultation features). Second, user experience indicators that might positively influence information processing (i.e. satisfaction, involvement, perceived relevance, perceived active control, perceived cognitive load) were measured to get insight in how patients in the experimental condition experienced the Patient Navigator. Third, the Patient Navigator was evaluated by comparing patients in the control condition and patients in the experimental condition on patient outcomes related to the consultation; anxiety levels before and after the consultation, participation during the consultation, satisfaction with the consultation and recall of information given during the consultation. Since the Patient Navigator was especially developed with the older patient in mind, the role of age was also taken into account for each of these measures.

To get insight into the user experience indicators and patient outcomes related to the consultation, patients in the experimental condition filled out questionnaires at different moments after using the Patient Navigator and surrounding the consultation with the surgeon. The first questionnaire was administered two days before the consultation, after using the Patient Navigator (T1a). On the day of the consultation with the surgeon, patients filled out the second questionnaire in the hospital right before the consultation (T1b), the consultations with the surgeon were recorded (T2a) and patients filled out the third questionnaire in the hospital right thereafter (T2b). The last questionnaire was administered two days later (T2c). Some measures were only included in one of the questionnaires, for example scales measuring stable, trait characteristics that were not expected to change over time (i.e. demographics, coping style, frailty, self-efficacy) or measures related to using the Patient Navigator or related to the consultation. User experiences were measured at the first measurement moment after using the Patient Navigator (T1a) and measures related to the consultation (i.e. satisfaction with the consultation and recall) were included in the questionnaire two days after the consultation (T2c), since right after the consultation (T2b) patients often had other appointments in the hospital, making this measurement moment not appropriate for measures that took longer to fill out. However, since anxiety is a situationally specific state concept and more temporary in nature [48], this measure was included in all questionnaires (T1a, T1b, T2b and T2c) to investigate if the effect of the Patient Navigator on anxiety varied over time. An overview of the measurement moments is presented in Table 1.

**Table 1:** Measurement moments

Questionnaire	Timing	Measures
T1a	2 days before the consultation (online) After using the Patient Navigator*	- Demographics - Psychosocial information - Coping style - User experience outcomes* - Satisfaction* - Involvement* - Perceived Cognitive Load* - Perceived Relevance* - Perceived active control* - Patient Outcomes related to the consultation - Anxiety
T1b	Right before the consultation (in the hospital)	- Patient Outcomes related to the consultation - Anxiety
T2a	Consultation recording	- Patient Participation outcomes - Absolute contribution - Relative contribution - Questions and assertions
T2b	Right after the consultation (in the hospital)	- Psychosocial information - Frailty - Patient Outcomes related to the consultation - Anxiety
T2c	2 days after the consultation (online)	- Psychosocial information - Self-efficacy - Patient Outcomes related to the consultation - Anxiety - Satisfaction with the consultation
T2c Recall	2 days after the consultation (via phone)	- Patient Outcomes related to the consultation - Recall

*Only measured in the experimental condition

This study was preregistered at Trialregister.nl, NTR5919, and received ethical approval by the Review Board of the Amsterdam School of Communication Research (2017-PC-7979) and the medical ethical review boards of the hospitals that participated in the study (METC-nr: 13-061).

### Participants and procedure

Participants were patients who were diagnosed with colorectal cancer in one of the four participating hospitals. Patients were awaiting further information regarding their diagnosis (i.e. stage of their cancer and possible metastases) and treatment options. This information would be given during a consultation with a surgeon, as part of usual care. Patients that were scheduled for this consultation and met the inclusion criteria (sufficient command of the Dutch language, able to read, no cognitive impairment according to their medical record, and access to the internet) were approached to participate in the pilot RCT. Once the consultation was scheduled, patients were asked by a nurse or medical secretary if they wanted to receive information about the study. The recruitment phase lasted from April 2018 through February 2020. Data collection was ended because of the COVID-19 crisis. During this period, the contact information of 162 patients that were suitable candidates and agreed on being contacted about the study was passed on by the hospital staff. Not all patients were successfully contacted (because of technical difficulties, e.g. wrong phone numbers, or because they did not met the inclusion criteria upon closer inspection), resulting in 141 patients that were approached. Informed consent was obtained from all the patients and healthcare providers.

**Fig. 2:**
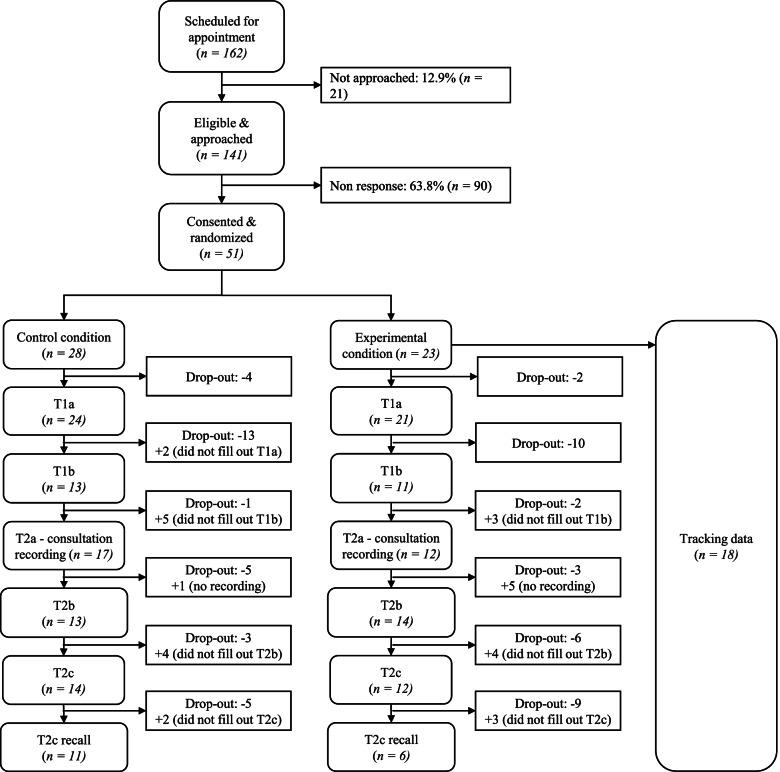
Flowchart of participant inclusion and drop-out

Patients willing to receive further information were randomly assigned to either the control condition and received the usual care procedure without any alterations by the researchers, or to the experimental condition where they received the Patient Navigator in addition to the usual care procedure. Randomization was done before patients consented to participate because in the experimental condition the informed consent also included information about the tracking data. No later than two days before the scheduled consultation, interested patients were called by the study coordinator to explain the study and what participation would entail. Interested patients received an informed consent form, the first online questionnaire (T1a) and, if allocated to the experimental condition, a personalized tracking link to the Patient Navigator, via e-mail. Patients allocated to the experimental condition were instructed to open the Patient Navigator as preparation for their consultation and complete the first questionnaire (in both the control and experimental condition). Patients had to visit the Patient Navigator by clicking on a personalized link, connected to a unique patient ID, provided to them via e-mail. Patients in the control condition were instructed to complete the first questionnaire before the consultation with the surgeon. Of the 141 patients that were approached to participate in the pilot RCT, 51 patients, seen by 17 surgeons, consented to participate, meaning an inclusion rate of 36.2%. Of the 51 patients, 28 were randomized to the control condition and 23 to the experimental condition and received the Patient Navigator. Of the latter 23 patients, 22 patients actually visited the Patient Navigator before the consultation (tracking data available or indicated they visited the Patient Navigator in T1a). As a result of non-response, 45 patients completed questionnaire T1a, 24 completed questionnaire T1b, 27 completed questionnaire T2b, 26 completed questionnaire T2c, 17 answered the recall questions in T2c and 29 consultations were recorded. Non-response analyses showed there were no differences between patients who consented to participate and patients who did not consent regarding age (*F* = .621, *p* = .432) and gender (*F* = .006, *p* = .938). See Fig. 2 for an overview of the inclusion process, non-response, and drop-out.

### Measures

#### Background variables

Socio-demographic information was obtained in the first questionnaire with questions regarding age, gender, education level, living situation and internet usage. Three categories were formed for education level (low, middle and high). Besides, coping style was measured, since individuals can differ in their tendency to seek or avoid information when coping with threatening medical situations [49, 50]. Three items addressed monitoring intentions (i.e. intentions to search for information) about their medical situation ranging from 1 (‘not at all applicable to me’) to 5 (‘very much applicable to me’). Higher scores mean higher monitoring coping style (*α* = .89). Frailty, or age-related decline in physical, cognitive, social and psychosocial functioning, was measured with the Groningen Frailty Indicator (GFI, [51], consisting of 15 items with scores ranging from 0 – 15. Higher scores indicate higher frailty levels. Lastly, self-efficacy, or an individual’s beliefs regarding communicating with a healthcare provider, was measured with a short version (five items) of the Patient-Physician Interactions Questionnaire [52]. Answers ranged from 1 (very confident) to 5 (not confident at all). A sum score was calculated and scores ranged from 15 to 25, with higher scores indicating higher self-efficacy levels.

#### Patient Navigator Usage

##### Usage of the Patient Navigator

Use of the Patient Navigator was monitored with a built-in tracker that measured the activity of the patients on the Patient Navigator like time spent in total and time spent on each separate page, number of visits, number of clicks, if the patient self-tailored the Patient Navigator and if the patient watched video’s.

#### Patient Navigator Experience Outcomes

##### Satisfaction with the Patient Navigator

Satisfaction with the Patient Navigator was measured with a 10-item website satisfaction scale [32, 39], consisting of subscales assessing the attractiveness (*α* = .93), comprehensibility (*α* = .99 ) and emotional support patients experienced from the Patient Navigator (*α* = .98). Patients answered on 7-point Likert scales ranging from 1 (totally disagree) to 7 (totally agree), with higher scores indicating higher satisfaction levels.

##### Involvement with the Patient Navigator

Involvement with the Patient Navigator was assessed with a website involvement scale [53], comprising five items with answer options ranging from 1 (totally disagree) to 7 (totally agree) (*α* = .91), with higher scores meaning more involvement.

##### Perceived Cognitive Load

The perceived cognitive load, of the Patient Navigator was measured with two items [54]. Items addressed the used amount of cognitive resources, for example ‘viewing the information on the Patient Navigator caused me a lot of effort’ and ranged from “totally disagree” (1) to “totally agree” (7) (*r* = .95, p < .01) with higher scores indicating higher perceived cognitive load.

##### Perceived Active Control

Patients’ perceived active control over the Patient Navigator was evaluated with a four item, 7-point Likert scale (α = .96; [55]. Items measured the patient’s beliefs regarding their influence over the information provided in an online environment, for example ‘I felt that I had a great deal of control while visiting the Patient Navigator’. Items ranged from 1 (totally disagree) to 7 (totally agree), with higher scores meaning more perceived active control.

##### Perceived Relevance

The perceived relevance, or how relevant patients experienced the information in the Patient Navigator with regards to their own situation, was assessed by means of a three item scale [56, 57], with items as ‘The tool seemed especially made for me’, Answer options ranged from 1 (totally disagree) to 7 (totally agree) (*α* = .72). Higher scores indicate more perceived relevance.

#### Patient Participation Outcomes

The consultations patients had with surgeons were audio recorded, directly after the consultation transcribed by research assistants and coded by research assistants or the researcher on patient participation using three measures. Patient’s absolute contribution to the consultation was indicated by the absolute amount of words used by the patient. Their relative contribution to the consultation was assessed with a percentage score specifying the ratio of words used by the patient divided by the number of words used by the surgeon [58]. Patient’s contextual contribution was measured with the number of questions and assertions expressed by them during the consultation. These questions and assertions were coded as such when they regarded the patient’s illness or related issues such as treatment options [59]. For this contextual contribution, ten percent was coded by a second independent coder resulting in a sufficient intercoder reliability (*κ* = .57, *p* < .03, [60]. More words, higher relative contribution and higher contextual contribution mean more patient participation.

#### Patient Outcomes related to the Consultation

##### Anxiety

Anxiety was measured at all measurement moments (T1a, T1b, T2b & T2c) with the short Dutch version of the State-Trait Anxiety Inventory (STAI-6) [48]. Patients rated on a 4-point scale to which degree they felt anxious in that moment. Answer options ranged from 1 (not at all) to 4 (very much), with higher scores pointing at higher levels of anxiety (T1a: *α* = .69; T1b: *α* = .66; T2b: *α* = .66; T2c: : *α* = .70).

##### Satisfaction With Consultation

Patient satisfaction with the consultation was measured with a five item scale (PSQ, [61], ranging from 1 (‘not satisfied at all’) to 5 (‘completely satisfied’) (*α* = .91), with higher score indicating higher satisfaction levels.

##### Information Recall

Information recall was measured based on transcribed audio recordings and the NPIRQ (Dutch version of the Patient Information Recall Questionnaire [43]. According to this method, questions were composed for each patient based on the transcripts of the consultations patients had with surgeons. Correct answers to these questions originated from these transcripts as well because they were statements made by the surgeon during the consultation. Patient could answer the questions with "this information was not discussed", "this information was discussed, but I can't remember the details", and “this information was discussed, namely …”. The first two answer options resulted automatically in a score of 0. If patients answered with the latter, the answer was coded based on correspondence with the utterance of the surgeon and could result in a score of 0 (not recalled correctly), 1 (partially recalled) or 2 (completely recalled). These scores were added up into a sum score. A percentage was calculated regarding the obtained recall score (range 29.2% - 100%) relative to the maximum achievable score [43], with higher scores meaning better information recall. A second independent coder coded 10% of the cases for intercoder reliability (mean *κ* = .985, *p* = .003) [60].

### Statistical Analyses

For the evaluation of the Patient Navigator descriptive analyses were carried out to provide insight into the observational data (i.e. tracking data regarding Patient Navigator usage and consultations) and self-reported satisfaction measures (i.e. satisfaction in terms of attractiveness, comprehensibility and emotional support, involvement, perceived cognitive load, perceived active control and perceived relevance). ANOVA’s were conducted to check the distribution of demographic variables (i.e. age, gender, education level, living situation), psychosocial information (coping style), internet usage, hospitals and healthcare providers over the conditions. Variables that were unequally distributed over the conditions were controlled for in further analyses. Descriptive analyses were conducted regarding the Patient Navigator’s usage and user experience outcomes. Frailty and self-efficacy were measured as background information as well, but due to missing data analyses were not reliable and are therefore not reported.

In addition, regression analyses were carried out to check whether usage and user experiences differed depending on patient’s age. For comparing patients who received the Patient Navigator and patients who did not, ANOVA’s regarding the outcome measures (anxiety levels before and after the consultation, satisfaction with the consultation, patient participation during the consultation and recall of information provided during the consultation) were conducted. Age differences were supposed to be checked by incorporating age as a moderator in the ANOVA analyses, however due to the small sample size this was not possible.

## Results

### Sample and Randomization Check

Patients that filled out T1a ranged in age from 52 to 89 years (*M* = 69.07, *SD* = 9.77) and 53.3% was male. About one third (31.1%) of the patients had received lower education (primary education or lower vocational education), 26.7% medium education (secondary vocational education) and 42.2% had a higher education level (higher professional education or scientific education). Most patients live together with their partner (60%). On average, patients spend 7.42 hours on the internet per week (*SD* = 7.85, range 0 – 40). Background information of the patients is given in Table 2.

**Table 2:** Sample characteristics

Background variables	Experimental condition	N	Control condition	N	Total sample	N
**Demographic information**		**21**		**24**		**45**
Age (years), mean (SD)*	66.10 (7.75)	21	71.67 (10.74)	24	69.07 (9.77)	
Gender***		***21***		***24***		***45***
Male, n (%)	15 (71.4%)		9 (37.5%)		24 (53.3%)	
Female, n (%)	6 (28.6%)		15 (62.5%)		21 (46.7%)	
***Education level***		***21***		***24***		***45***
Low, n (%)	7 (33.3%)		7 (29.2%)		14 (31.1%)	
Medium, n (%)	6 (28.6%)		6 (25%)		12 (26.7%)	
High, n (%)	8 (38.1%)		11 (45.8%)		19 (42.2%)	
**Average time spent on internet (hours per week)**		**21**		**22**		**43**
In hours, mean (SD)	8.57 (6.98)		6.32 (8.62)		7.42 (7.85)	
**Psychosocial information**						
Coping style^1^, mean (SD) (T1a)**	3.73 (1.00)	21	2.94 (1.15)	21	3.33 (1.14)	42
**Living situation**		**21**		**24**		**45**
Alone, n (%)	1 (4.8%)		7 (29.2%)		8 (17.8%)	
With partner, n (%)	14 (66.7%)		13 (54.2%)		27 (60%)	
With children, n (%)	1 (4.8%)		1 (4.2%)		2 (4.4%)	
With partner and children, n (%)	4 (19%)		2 (8.3%)		6 (13.3%)	
Other, n (%)	1 (4.8%)		1 (4.2%)		2 (4.4%)	

1. Measured on a 5-point Likert scale.

* Significant, p < .10 ** Significant, p < .05

***Significant, p < .01

Patients in the experimental condition did significantly differ regarding gender (*F* = 5.594, *p* = .023) and coping style (*F* = 5.66, *p* = .022) from patients in the control condition, in such a way that participants in the experimental condition where on average younger and a higher monitoring coping style than participants in the control condition. There were no differences between the conditions regarding age (*F* = 3.880, *p* = .055), education level (*F* = .211, *p* = .648), living situation (*F* = 2.622, *p* = .113) and internet usage (*F* = .883, *p* = .353), healthcare provider (*F* = .061, *p* = .806) and hospital (*F* = .310, *p* = .581)

Gender and coping style were included as control variables for further analyses if they significantly correlated with the outcome measure. Gender did not correlate with any of the outcome measures (i.e. patient participation measures, anxiety, satisfaction and recall) and was therefore left out as control variable in further analyses. Coping style did significantly correlate with some of the outcome measures and was thus taken into account. Lastly, even though hospital and surgeon did not significantly differ across the conditions, both were significantly correlated to outcomes and were therefore also taken into account as control variables for further analyses.

### Usage of the Patient Navigator

Out of the 23 patients that were assigned to the experimental condition, 18 patients visited the Patient Navigator before their consultation via the tracking link they received from the researcher. Another four patients indicated they visited the Patient Navigator, but no tracking data is available for these patients because they did not access the Patient Navigator via the personalized tracking link. One patient only visited the Patient Navigator after the consultation and was excluded from further analyses, resulting in data of 18 patients available for analyses. These patients spent on average 17 minutes and 58 seconds browsing through the Patient Navigator (*SD* = 30:35; range: 1:52 min – 135:26 min). Half of the patients visited the Patient Navigator more than once before their consultation (50.0%), but only one fifth of the patients visited the Patient Navigator more than twice (22.2%). Approximately one fifth (21.1%, 4/19) of the patients visited the Patient Navigator after their consultation as well (*M* = .37, *SD* = .83, range number of visits = 0 – 3). The average amount of clicks was 62.44 (*SD* = 62.45; range: 6 – 275). Out of the six main pages on the Patient Navigator, patients visited on average 3.94 pages (*SD* = 1.83), ranging from 1 to 6 pages.

Patients mostly visited the ‘Treatment’ (94.4%, 17/18) and ‘Diagnosis’ page (83.3%, 15/18). Although five patients clicked on the self-tailoring page, three patients actually tailored the information presentation mode or content. Only one patient played two of the animations. Almost half of the patients used the QPL feature, while only one patient used the audio-facility feature. An overview of the Patient Navigator usages is presented in Table 3. A table showing more detailed usage patterns is included as appendix 4 Table 6.

**Table 3:** Usage of the Patient Navigator

USAGE variables	outcomes (n=18)
**Time spent on Patient Navigator**	
Total time spent (mm:ss), mean (SD)	17:58 (30:35)
**Total number of visits**	
Mean (SD)	1.94 (1.35)
**Total number of clicks**	
Mean (SD)	62.44 (62.45)
**Total number of Visits Main Pages**	
Mean (SD)	3.94 (1.83)
**Web pages, n (%)**	
Treatment	17 (94.4%)
Diagnosis	15 (83.3%)
Aftercare	13 (72.2%)
About Colorectal Cancer	11 (61.1%)
Practical tips	10 (55.6%)
Contact	5 (27.8%)
**Watched at least one video**	
n (%)	1 (5.6%)
**Usage of pre- and post-consultation features, n (%)**	
Prepare for consultations (QPL)	8 (44.4%)
Listen back to consultation (audio-facility)	1 (5.6%)
**Self-tailoring, n (%)**	19 (21.1%)
Navigated to self-tailoring page	5 (27.8%)
Actually engaged in self-tailoring	3 (16.7%)

#### Age differences in usage of the Patient Navigator

Time spent on the Patient Navigator was influenced by age (*β* =2.65, *p* = .053), in such a way that older patients spent more time on the Patient Navigator. Regarding number of visits, page visits, and clicks, no differences were found depending on age.

#### Evaluation of the Patient Navigator (n=18)

Patients were most satisfied with the comprehensibility of the Patient Navigator (*M* = 6.04, *SD* = 1.49), followed by the attractiveness of the Patient Navigator (*M* = 5.44, *SD* = 1.21) and emotional support patients experienced from the Patient Navigator (*M* = 4.53, *SD* = 1.66). On average, patients were moderately involved with the Patient Navigator (*M* = 4.52, *SD* = .1.42) and perceived the Patient Navigator as moderately relevant (*M* = 4.52, *SD* = 1.32). The perceived cognitive load of the Patient Navigator was low (*M* = 2.29, *SD* = 1.65), while active control was perceived as relatively high (*M* = 6.38, *SD* = .77).

#### Age differences in Evaluation of the Patient Navigator

No differences depending on age were found regarding satisfaction with the comprehensibility (*β* =.345, *p* = .148), satisfaction with the attractiveness (*β* =.294, *p* = .222), satisfaction with the emotional support (*β* =.016, *p* = .947), involvement (*β* =.622, *p* = .542), cognitive load (*β* =1.279, *p* = .218), active control (*β* =-1.570, *p* = .135), and perceived relevance (*β* =-.780, *p* = .446).

### Pilot RCT Effect Outcomes

#### Anxiety

**Fig. 3:**
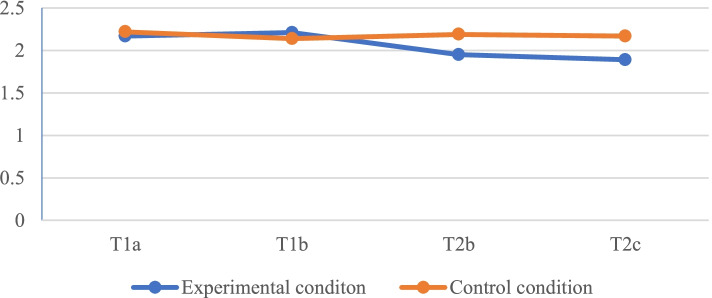
Anxiety levels across measurement moments

Results showed no significant difference in anxiety between patients in the control condition and patients who received the Patient Navigator two days before the consultation (T1a: *F* = .14, *p* = .71), right before the consultation (T1b: *F* = .19, *p* = .67) and right after the consultation (T2b: *F* = 1.98, *p* = .17). However, there was a significant difference between patients in the control condition and patients in the experimental condition regarding anxiety two days after the consultation (T2c: *F* = 4.46, *p* = .045) in such a way that patients who had used the Patient Navigator were less anxious than patients who had not (see Fig. 3).

#### Satisfaction with the Consultation

There was no significant difference between patients who received the Patient Navigator and patients who did not in terms of satisfaction with the consultation (*F* = .11, *p* = .74).

#### Patient Participation

Analyses showed a marginally significant difference in number of words used by the patient during the consultation between the control condition and the experimental condition (*F* = 2.97, *p* = .097). Patients who received the Patient Navigator used less words (*M* = 425.42, *SD* = 280.87) than patients that did not receive the Patient Navigator (*M* = 735.93, *SD* = 435.49). There was no significant difference between patients in the experimental condition and the control condition in relative contribution of the patient during the consultation (*F* = 1.95, *p* = .18) and number of questions and assertions of the patient (*F* = .00, *p* = .96).

#### Information Recall

There was no significant difference between patients in the experimental condition and patients in the control condition regarding recall of information provided during the consultation (*F* = .29, *p* = .60). For an overview of the means per condition see Table 4.

**Table 4:** Means and Standard Deviations of outcome variables per condition & total sample

Outcome Variables	Experimental condition	N	Control condition	N	Total sample	N
**PN Self-reported evaluation outcomes**		**18**				
***User Experience Outcomes***						
Satisfaction^1^ (total), mean (SD)	5.28 (1.24)					
Satisfaction with comprehensibility^1^, mean (SD)	6.04 (1.49)					
Satisfaction with attractiveness^1^, mean (SD)	5.44 (1.21)					
Satisfaction with emotional support^1^, mean (SD)	4.53 (1.66)					
Involvement^1^, mean (SD)	4.52 (1.42)					
Perceived relevance^1^, mean (SD)	4.52 (1.32)					
Perceived cognitive load^1^, mean (SD)	2.29 (1.65)					
Perceived active control^1^, mean (SD)	6.38 (.77)					
**Patient Participation outcomes**^**1**^		**12**		**17**		**29**
Duration consultation (mm:ss), mean (SD)	18:46 (10:30)		20:12 (7:13)		19:45 (8:17)	
Amount of words patient and surgeon combined, mean (SD)**	2601.50 (878.56)		3382 (1038.52)		3059.17 (1035.70)	
Amount of words patient, mean (SD)*	425.42 (280.87)		675.17 (442.19)		571.83 (397.99)	
Relative contribution - patient, % (SD)	16.52% (9.04)		21.33% (8.79)		19.2% (9.34)	
Relative contribution - surgeon, % (SD)	83.48% (9.04)		78.66% (8.79)		80.8% (9.34)	
Number of questions patient, mean (SD)	7.17 (7.27)		6.47 (5.89)		6.76 (6.38)	
Number of assertions patient, mean (SD)	1.00 (1.28)		1.65 (1.56)		1.38 (1.52)	
Contextual contribution (number of questions and assertions), mean (SD)	8.17 (7.72)		8.12 (6.40)		8.14 (6.84)	
**Patient outcomes related to Consultation**^**1**^						
Anxiety T1a^2^, mean (SD)	2.17 (.42)	21	2.22 (.53)	21	2.19 (.47)	42
Anxiety T1b^2^, mean (SD)	2.21 (.37)	11	2.14 (.42)	13	2.17 (.39)	24
Anxiety T2b^2^, mean (SD)	1.95 (.44)	14	2.19 (.45)	13	2.07 (.45)	27
Anxiety T2c^2^, mean (SD)* ^2^	1.89 (.27)	12	2.17 (.38)	14	2.04 (.36)	26
Satisfaction with consultation^1^, mean (SD)	4.43 (.47)	12	4.50 (.78)	13	4.47 (.64)	25
Information recall, mean (SD)	61.60 (22.41)	6	67.49% (24.70)	13	65.52% (23.47)	18

*Significant, p < .10

**Significant, p < .05

***Significant, p < .01

1. Measured on a 7-point scale

2. Measured on a 4-point scale.

## Discussion

### Review of main findings

The aim of this study was to develop, implement and evaluate the Patient Navigator an online health information tool for older colorectal cancer patients using the MRC Framework [24]. Based on a carefully designed series of preliminary studies, we successfully developed the Patient Navigator that was positively evaluated in terms of user experience outcomes (i.e. satisfaction, involvement, perceived cognitive load, perceived active control and perceived relevance). Noticeably, usage of the Patient Navigator led to lower anxiety levels after the consultation and appeared to reduce patient participation in terms of word use by the patient.

Regarding usage of the Patient Navigator, usage patterns point out that patients used the Patient Navigator more to prepare for the consultation instead of checking for information after the consultation. Patients visited the ‘treatment’ and ‘diagnosis’ pages, containing information to prepare for consultations, the most, as compared to other pages that might be more relevant after the consultation for example about what might follow after the end of treatment. To stimulate usage of the website after the consultation, it might have been helpful if healthcare providers actively promoted the website during the consultations with patients, and specifically point to pages that might be relevant later on during treatment and/or follow-up. In addition, the QPL function to prepare for consultations was used more often than the function to listen back to the consultations. Apparently, patients are not inclined to listen back to their consultations on their own initiative. However, in view of the effectiveness of audio-facilities in improving patients’ uptake of information [42], it can be argued that patients should be encouraged to use such audio-facility functions. In the current study, post-consultation features of the Patient Navigator, including the audio-facility, were possibly insufficiently introduced to patients. As a result, even if patients experienced the need for more information after the consultation, the Patient Navigator might not have come to mind as a relevant tool.

Contrary to our expectations, only one quarter of the patients visited the self-tailoring page and only three patients actually self-tailored the content or information presentation mode. Based on the outcomes of the preparatory think aloud usability study, patients in the pilot RCT who entered the Patient Navigator via the personalized link, were presented with a default mode showing all information and all information presentation modes. The limited use of the self-tailoring option might in hindsight be explained by choosing for this default mode, since self-tailoring would imply decreasing the amount of information and/or decreasing the number of modalities in which the information was presented. In previous comparable studies in which self-tailoring meant an expansion of information or modalities, more patients engaged in self-tailoring [36]. Even though the results of the think aloud usability study showed patients prefer to start with all information and information presentation modes, according to previous research an online tool showing all available information presentation modes was less effective in terms of perceived active control, perceived relevance, involvement, cognitive load, satisfaction with the tool and recall of information when compared to when the information presentation mode in the tool was self-tailored [37]. This points out a discrepancy between what patients prefer and what is most effective regarding evaluative and cognitive outcomes.

Lastly, only one patient watched a video. This is noteworthy since the default mode of the Patient Navigator included videos. In contrast, in a previous study regarding self-tailoring information presentation modes among a similar patient population, almost a third of the patients watched the videos [36]. These were mostly patients who had selected videos as information presentation mode when self-tailoring. Since in the current study only a few patients engaged in self-tailoring the information presentation mode, this might explain why the videos were not watched by more patients. Our results imply that patients deliberately choosing for a specific mode can feel activated to use the information presented in their mode of choice, whereas without such self-tailoring, they might be less likely to use their preferred information presentation mode. This is a missed opportunity as previous research showed videos and animations were effective in increasing satisfaction and information recall [28, 62]. Again, this shows that preference and effectiveness are not in accordance. Similar mismatches between preferences and effectiveness are found for visual aids portraying health information [63, 64]. The conflicting results in the current study raises the question whether designs for online health information should be based on patients’ preferences or on effectiveness regarding patient outcomes. Even though designs that are appreciated are not automatically more effective in terms of patient outcomes, previous research did show that satisfaction with the design of online health information is an important predictor of information recall [26]. Additionally, for older adults satisfaction with online health information designs increases motivation to process the information [28, 32]. It is therefore advised to strive for online health information designs that have proven to be effective in terms of patient outcomes, while also taking into account patients’ preferences.

Satisfaction, involvement, and perceived cognitive load of an online tool are argued to be relevant user experience outcomes in stimulating information processing and positively influencing cognitive patient outcomes [28]. Furthermore, for a tool allowing patients to self-tailor the information presentation mode, perceived relevance and perceived active control have shown to positively influence satisfaction with the tool and cognitive patient outcomes [37]. The results of the current study extend these findings to anxiety, and patient participation outcomes, specifically the amount of words used by the patient. Thereby, these results support the importance of patients’ user experiences with an online health information tool in the effectiveness of that tool.

While the information presented in the Patient Navigator was specifically selected for colorectal cancer patients, patients experienced the Patient Navigator as moderately relevant. A previous comparable study pointed out that especially patients that engaged in self-tailoring perceived the information as highly relevant [36]. Therefore, the lack of self-tailoring by patients in the current study could have caused the lower perceived relevance scores, stressing even more that the lack of self-tailoring was a missed opportunity and could have been stimulated more.

Luckily, perceived active control of the Patient Navigator was high, which is an important factor in increasing satisfaction with a tool [37]. Perhaps the possibility to self-tailor the Patient Navigator was already enough to increase active control, despite the fact that self-tailoring was not often applied by patients. However, taking into account previous research, actually self-tailoring could have led to even higher levels of perceived active control [37].

Surprisingly, patients that received the Patient Navigator used marginally significantly less words during the consultation and the total amount of words used during the consultation by both the patient and the surgeon was marginally significantly lower as compared to the control condition. In line, the average total duration of the consultation showed a trend towards shorter consultation time in the experimental group as compared to the control group. Based on these results, we carefully conclude that less words were needed during the consultations by both the patient and the surgeon if patients used the Patient Navigator, because some patient information needs were already fulfilled before the consultation, resulting in shorter consultations.

Opposing this finding, a previous study among a comparable patient population, found that newly diagnosed colorectal cancer patients who engaged in seeking online health information on their own initiative before a consultation with a surgeon, used more words during that consultation [14]. As an explanation for these contradictory findings, we speculate that if patients search for online health information independently this might result in confusion and worry, leading to patients experiencing a higher need for information during the consultation and therefore using more words. However, if patients receive appropriate information that is tailored to their situation and is provided by a reliable source this might result in a lower need for information during the consultation and patients might therefore use less words. Since the Patient Navigator covered many topics that would generally be discussed during consultation, patients in the current study may have used less words because some of their questions before the consultation had already been answered. Shorter consultations as a result of using the Patient Navigator, without impairing patient’s satisfaction, might be convenient for surgeons, given the limited amount of time they have available per consultation. Hence, future research could investigate if patients experience shorter consultations positively when relevant information has already been covered in an online preparatory tool.

Patients who received the Patient Navigator asked just as much questions and expressed a comparable amount of assertions compared to patients who did not receive the Patient Navigator. This was unexpected because according to previous research, pre-consultation features such as a QPL function contributes to patients asking more questions [65]. However, in the current study, only less than half of the patients in the experimental condition used the QPL function, which could explain the absence of an effect on patients’ questions and assertions during consultations. In addition, patient who used the QPL function could have already received an answer from the Patient Navigator if more general questions were selected in the QPL, on which a computer-generated answer was provided. This could have made it unnecessary for patients to ask certain questions during consultations.

Contrary to our expectations, the Patient Navigator did not succeed in lowering anxiety levels before the consultation, increasing satisfaction with the consultation and improving information recall. However, patients who used the Patient Navigator reported lower anxiety levels two days after the consultation as compared to patients in the control condition. Previous research of our group showed that anxiety levels in colorectal cancer patients decrease especially after receiving a diagnosis accompanied by a curative treatment plan [50]. The results in the current study seem to point at a decrease in anxiety over time as well, with the highest anxiety levels two days before the consultation and the lowest at two days after the consultation. Usage of the Patient Navigator seems to decrease anxiety even stronger when time passes, resulting in a significant difference between anxiety levels of patients in the control condition and the experimental condition two days after the consultation. This is an interesting finding since some research has pointed out that patient outcomes such as lowered anxiety could lead to better health outcomes [71]. Future research could investigate whether patient outcomes influenced by online health information, such as anxiety, positively affect health outcomes, for example postoperative results.

Moreover, in reducing cancer patients’ anxiety, fulfilling cognitive and affective needs can play an important role [66, 67]. The main goal of the Patient Navigator was to provide patients with information as an attempt to reduce anxiety by fulfilling patients’ cognitive needs [15, 68]. However, anxiety is an emotion, and can therefore, especially be reduced by emotional support [66, 69], for example through sharing ones current or previous illness or treatment status with other patients online [70]. The Patient Navigator did not offer patients such a function, possibly resulting in the relatively low satisfaction with the emotional support (when compared to overall satisfaction) provided by the website, which in turn could explain the absence of an effect on anxiety levels before the consultation. For the development of similar online tools, addressing patients’ affective needs could be considered. For example, by implementing a discussion page, allowing patients to share their status or experiences in a peer 2 peer format.

### Strengths and Limitations

To the best of our knowledge, this study was the first to investigate actual usage and effectiveness of an online health information tool including multiple information presentation modes, self-tailoring options and pre- and post-consultation features in a clinical population. A major strength of this study is that the Patient Navigator was evaluated based on data collected during the diagnostic phase (i.e. from days before the consultation with the surgeon to during the consultation and days after the consultation). This longitudinal data is valuable in terms of ecological validity and exceptional because we succeeded in evaluating the use of the Patient Navigator at a time where patients probably feel a lot of uncertainty and experience the highest information needs. Another strength worth mentioning is that the Patient Navigator was evaluated based on a combination of observational (i.e. usage of the Patient Navigator and recordings of the consultations with surgeons) and self-reported data, again contributing to the ecological validity of the results. The method of collecting usage data via a built-in tracking system in the Patient Navigator is innovative. The multicentre approach, collecting data in multiple hospitals distinguishes this study from previous research and is beneficial for the generalizability of our findings.

Approximately one third of the patients that were approached decided to participate in the study, which is satisfactory in view of the large emotional burden because of the diagnosis they had recently received. However, the sample size and the relatively high drop-out rate brings several limitations. First of all,with regards to statistical power. Therefore, it cannot be ruled out that the absence of an effect of the Patient Navigator on consultation duration and patient participation in terms of relative contribution and information recall is due to the lack of statistical power. We wish to point out that the results of the current study, especially the comparisons between the control and the experimental conditions, should be interpreted with considerable caution. The drop-out after each questionnaire contributed even further to this limitation, which is why some analyses with regards to dependent variables measured in one of the later measurement moments could not be carried out. Additionally, the small sample size hampered analysing differences in the effectiveness of the Patient Navigator between older and younger patients.

Possible voluntary bias is another limitation of this study. Even though non-response analyses revealed patients who participated in the study did not differ from patients who did not participate in terms of age, more frail or vulnerable patients might be underrepresented in this study as they may have declined being contacted about this study because they experienced it as too much of a burden. In addition, we might not have been able to include patients that cope with the situation by avoiding information about their illness because they were probably less willing to participate in a study where they would receive information.

Another methodological limitation of the current study is that patient participation was operationalized as a quantitatively measured concept. Even though usage of the Patient Navigator might not have led to asking more questions, it could have been the case that patients who used the Patient Navigator asked questions about different topics than patient who did not use the Patient Navigator. However, due to the operationalization of patient participation we were not able to gain insight into the type of questions asked by patients in both conditions. Future research is advised to look into the effects of an online health information tool on patient participation in a qualitative manner, by for example coding the topics that are addressed in patients’ questions.

Lastly, due to the design of the current study it cannot be said with certainty which features of the Patient Navigator contributed most to the positive evaluations and the effects of the Patient Navigator. Even though the studies conducted in the development and pilot testing phase provided insight into whether features were appreciated by patients or not, we can only speculate about which features actually contributed to the evaluation and effectiveness of the final version of the Patient Navigator. Researchers interested in further optimalization of the design of online health information tools are advised to conduct studies testing different versions of the same tool to find out which features are most responsible for positive outcomes and which features could be excluded without compromising this.

### Conclusions

Overall, the Patient Navigator has the potential to positively influence patients’ experiences during the diagnostic phase. The Patient Navigator was well used and received positive evaluations of patients regarding satisfaction, involvement, perceived cognitive load, and perceived active control, possibly explaining the effects found on patient participation and anxiety.

The effects of using the Patient Navigator on patient participation and anxiety hold promising implication for healthcare providers. The decrease in the amount of words used by both the surgeon and the patient during consultations, resulting in shorter consultations, introduces the possibility to use the time reserved for the consultation to address other patient needs. For example, patients could be asked if there are certain topics they want to discuss more in depth instead of only focusing on standard information regarding the diagnosis and treatment. The decrease in anxiety levels after the consultation may imply the patient being in a more comfortable position while awaiting the treatment.

Therefore, based on the results of the current study, it is advised to develop, test and implement online health information tools with a particular focus on presenting information in multiple modalities, including self-tailoring options and including features to prepare for consultations with healthcare providers. Post-consultation features could be considered, but based on the results of the current study, these features should be thoughtfully introduced to patients. The positive results of the Patient Navigator in terms of evaluative outcomes, patient participation and anxiety show the potential of such online health information tools and can be used as input for the development of similar tools.

## Data Availability

All data on which the conclusions were based were included in the main paper. The full datasets used are available on request. Request for the full datasets can be directed to the corresponding author.

## Appendix 1

### Method of the usability study

Think-aloud observations for seven available online health information tools were video-recorded. The panel consisted of fifteen colorectal cancer patients (or survivors of colorectal cancer) and their partners (n=8) of at least 70 years old. Participants were asked to think aloud while they were performing different search, application and evaluation tasks within the environment of the online cancer information tools, using an interview protocol for each of the three separate tasks. For the search tasks patients were asked to imagine a scenario and search for information relevant for them given the scenario. Patients needed to search for this information without any instructions regarding where to find it, to check how participants spontaneously used the online tools as well as to assess what information participants would search for in specific situations. During the application tasks, participants received specific instructions to navigate to a certain page, which provided more information about the general navigation structure of the online tool. By means of evaluation tasks the participants provided their opinion about the content and usefulness of the tools.

## Appendix 2

### Method of the lay-out study

Twenty-two colour schemes were presented in different environments. First, patients were provided with screenshots of the PatientVoice, presented in 15 different colour schemes. Next, to check for more colour schemes independently of the lay-out of the PatientVoice, colour schemes were also presented in environments of seven other already existing websites. In addition, various visuals (including drawn illustrations and photos) were presented to participants in the form of visuals derived from the PatientVoice, as well as other visuals, resulting in a total of 58 visuals. Participants rated all colour schemes on contrast, readability, visibility and preference and were asked to select their favourite colour scheme and top three favourite visuals

## Appendix 3

Table 5

**Table 5:** Overview of phases and sub studies

Phase & sub study	Aim	Method	Results & insights	Implications for Patient Navigator
***1) Development:*** Think aloud study (Bolle et al., 2016)	Gain insight into usability issues and the perceived usefulness older cancer patients experience when using existing cancer-related online health information tools	Video-recorded think-aloud observations for 7 Web-based health information tools	- Patients appreciate and were able to use cancer-related online health information tools. - Patients had difficulties navigating through websites that had complex structures (eg, multiple navigation bars) and layouts that were inconvenient for example, buttons that were too small to click on). - Patients appreciated information presented in different modalities (mostly if it was to clarify the text and less for aesthetic reasons). - Patients varied greatly in terms of the amount of information they wanted to receive.	- Navigation structure and possibilities should be kept simple - Information should be concise - Amount of questions for QPL tool should be limited and patients should be provided with clear explanation about QPL. - Information should be presented in multiple modalities; videos and illustrations should be developed.
***1) Development:*** Lay-out study	Test different lay-out options among older cancer patients	Interviews	- Patients preferred contrasting color schemes - Patients preferred illustrations of characters instead of photos.	- Contrasting color schemes are advised - Illustrations of characters are advised.
***2) Pilot testing:*** Think aloud observations	Investigate how older adults and their partners evaluate the Patient Navigator	Think aloud observation while interacting with the Patient Navigator	- Overall patients were satisfied when using the Patient Navigator, but had some navigation problems Several usability problems were identified: - Patients had difficulties finding/or did not actively seek the decision support and consult preparation. - Patients had difficulties with adjusting the content of the tool to their personal situation	- Make labels and headers representative for their contents. - Make entire menu buttons clickable. - Rename the labels of the menu - Location of possibility to tailor the Patient Navigator should be clearer. Change name of the header. - Menu with explanation of structure on the homepage. - Health information should be checked by health professionals to ensure it is correct.
***2) Pilot testing:*** Usability study	Investigate how older adults and their partners evaluate the Patient Navigator on its usability (how effective, efficient and satisfied they are when using the Patient Navigator).	Usability questionnaire	- Patient Navigator was evaluated positively regarding the attractiveness, comprehensibility, emotional support and different usability issues. - Patients still experienced some difficulties with the labels and menu of the Patient Navigator.	- Renaming the labels in the menu again. - Adapting the lay-out of the menu shown on the homepage.

## Appendix 4

Table 6

**Table 6:** Detailed Usage Patterns

Background variables	outcomes (n=18)
***Web pages, n (%)***	
**Treatment**	17 (94.4%)
*Surgery Colon*	11 (61.1%)
*Surgery Rectum*	5 (27.8%)
*Surgery Small intestines*	4 (22.2%)
*Chemotherapy*	5 (27.8%)
*Targeted therapy*	4 (22.2%)
*Radiation*	5 (27.8%)
*Palliative Surgery*	10 (55.6%)
**Diagnosis**	15 (83.3%)
*Blood tests*	7 (38.9%)
*Coloscopy*	5 (27.8%)
*CT-Colonography*	5 (27.8%)
*CT-Scan*	5 (27.8%)
*Double balloon enteroscopy*	2 (11.1%)
*Duodenoscopy*	3 (16.7%)
*Echography*	3 (16.7%)
*Endo-Echography*	3 (16.7%)
*Geriatric Screening*	3 (16.7%)
*Lung Photo*	2 (11.1%)
*MRI-Scan*	4 (22.2%)
*Rectoscopy*	2 (11.1%)
*Sigmoidoscopy*	2 (11.1%)
*Video-Endoscopy*	3 (16.7%)
**Aftercare**	13 (72.2%)
*Clicked on Link of aftercare website*	2 (11.1%)
**About Colorectal Cancer**	11 (61.1%)
*Colon*	7 (38.9%)
*Rectum*	3 (16.7%)
*Small Intestines*	1 (5.6%)
**Practical tips**	10 (55.6%)
**Contact**	5 (27.8%)
***Watched at least one video***	
n (%)	1 (5.6%)
***Usage of pre- and post-consultation features, n (%)***	
**Prepare for consultations**	8 (44.4%)
*General questions for all treatments/tests*	6 (33.3%)
*Questions for specific treatment/test - QPL*	7 (38.9%)
*Used QPL*	6 (33.3%)
*Think about what is important*	3 (16.7%)
**Listen back to consultation (audio-facility)**	1 (5.6%)
***Self-tailoring, n (%)***	
Navigated to self-tailoring page	5 (27.8%)
Actually engaged in self-tailoring	3 (16.7%)
Tailored content	2 (11.1%)
Tailored Mode	3 (16.7%)
